# Papillary Thyroid Carcinoma Presenting As a Cystic Neck Lesion: Case Series

**Published:** 2018-01

**Authors:** Sethu T Subha, Mohd- Adzreil Bakri, Hisyam Salleh, Mohamad Doi, Abdul-Jalil Nordin

**Affiliations:** 1 *Department of Otorhinolaryngology,* * Faculty of Medicine & Health Sciences, University Putra Malaysia, * *Selangor,* * Malaysia* *.*; 2 *Department of Otorhinolaryngology, Hospital Serdang, Selangor, Malaysia*

**Keywords:** Cystic neck lesion, FNAC, Papillary thyroid carcinoma, 18 F-FDG-PET CT scan

## Abstract

**Introduction:**

Papillary thyroid carcinoma (PTC) constitutes 75–85% of all thyroid cancers. PTC usually presents as a subtle, commonly slow-growing, painless thyroid mass or a solitary nodule in the neck. This presentation of a cystic neck lump, without the presence of a thyroid nodule, may imitate the course of a benign disease, thus delaying diagnosis and proper treatment.

**Case Report:**

Three cases that had been initially presented as a cystic neck lesion in which a benign etiology was considered primarily were compiled in this study. PTC was only diagnosed after surgical excision of these cystic neck lesions in the first two cases, and after performing fine needle aspiration cytology (FNAC) and an 18fluorine-fluorodeoxyglucose positron emission tomography computed tomography (18F-FDG-PET CT) scan in the latter case.

**Conclusion:**

PTC can sometimes present as a cystic neck mass; a presentation which is usually related to a benign lesion. This case series emphasizes that patients who appear to have a solitary cystic neck mass must be treated with a high index of clinical suspicion. Although not a first-line imaging modality, 18F-FDG-PET can be extremely useful in assessing patients with a cystic neck lesion, where diagnosis is still uncertain after standard investigations such as ultrasonography and FNAC have been performed.

## Introduction

Papillary thyroid carcinoma (PTC) constitutes 75–85% of all thyroid cancers (1). The most common presentation for PTC is a thyroid mass; however, presentation as a solitary nodule or a cystic neck lump in cases of metastatic disease has also been reported (2–4). This presentation of a cystic neck lump (i.e. without the presence of a thyroid nodule) may imitate the course of a benign disease, therefore delaying diagnosis and proper treatment. In this case series, three patients presenting in a similar manner were selected as case studies. PTC was only diagnosed after surgical excision of these cystic neck lesions in the first two cases, and after performing fine needle aspiration cytology (FNAC) and an ^18^fluorine-fluorodeoxyglucose positron emission tomography computed tomography (^18^F-FDG-PET CT) scan in the latter case.

## Case Report

*Case 1*

**Fig 1:**
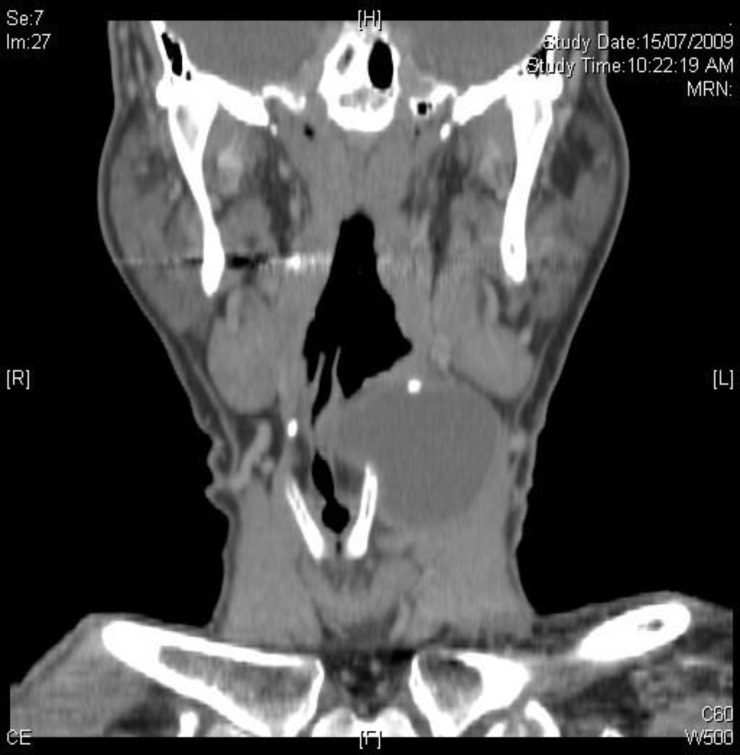
Coronal CT scan, demonstrating cystic lesion over the left side of the neck.

A 65-year-old Chinese gentleman with a background history of hypertension and glaucoma presented with a history of left neck swelling for few months, which had progressively been increasing in size. He had neither constitutional symptoms nor other complaints. Upon presentation, a lump measuring 8 × 5 cm at level II–III was visible on the left side of the neck. The lump was cystic, non-tender, and moved superiorly with swallowing but not with tongue protrusion. There were no other swellings on the neck. Flexible nasopharyngolaryngoscopy revealed slight fullness and medialization of the left pyriform fossa, with the epiglottis displaced to the right side.A computed tomography (CT) scan of the neck (Fig. 1) showed a cystic lesion over the left side of the neck, extending from the hyoid bone superiorly to the inferior border of the thyroid cartilage. The lesion lay adjacent to the left paravertebral space posteriorly and was limited by the body of the hyoid bone anteriorly. The hyoid bone was splayed, and its left horn was elevated by this lesion. The left carotid sheath and left sternocleidomastoid muscle were minimally displaced laterally by this cystic lesion. 

The fat plane between this cyst and the surrounding structure was clear. The mass was separated from a normal-looking homogenous and symmetrical thyroid gland. Other structures were reported as normal. FNAC of the mass showed mixed inflammatory cells, hemosiderin-laden macrophages, colloid, and cholesterol crystals that were suggestive of a colloid nodule with cystic degeneration.

Excision of the cyst was performed via a transcervical approach, and the finding was a well-encapsulated mass at level II measuring around 10 × 5 cm. The mass was medial and posterior to the sternocleidomastoid muscle, with a superior attachment to the hyoid bone and inferior attachment to the thyroid cartilage. 

The cyst ruptured during manipulation and spilled yellowish colloid fluid. Histopathological (HPE) findings described the sample as fibrotic wall fragments, with denuded surface epithelium in most areas, as well as ciliated columnar epithelium and squamous epithelium. 

The cyst wall showed mononuclear cell infiltrates and cholesterol cleft formation with foreign body giant cell reaction. Also seen was thyroid tissue adjacent to the cyst wall, and there was a focus of papillary carcinoma within the thyroid tissue. 

**Fig 2:**
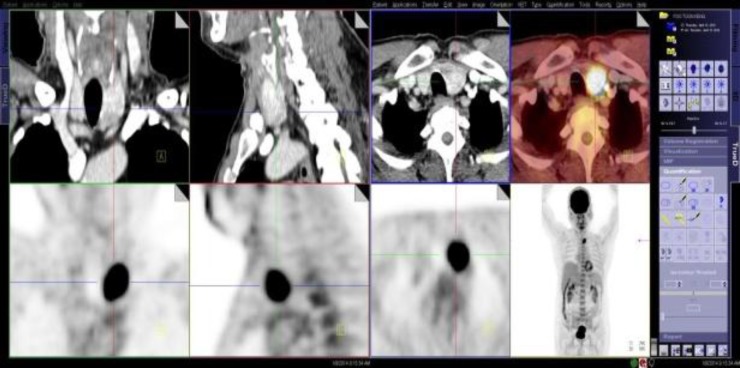
Multiplanar (coronal, sagittal and axial) and MIP images on CT and PET/CT (upper row) and on PET (lower row) demonstrating a subtle enlarged left thyroid lobe from a heterogenous enhancing lesion seen on CT. The lesion corresponds with high 18F-FDG intensity uptake

The tumor cells showed ground glass and grooved nuclei, and a papillary growth pattern. No lymphovascular invasion was found. An ^18^F-FDG-PET CT scan (Fig. 2) demonstrated a large focus of a high-intensity ^18^F-FDG uptake in the left thyroid lobe.

The patient underwent an endoscopic total left thyroid lobectomy and near total right thyroidectomy. HPE showed minimally invasive papillary carcinoma of the left thyroid lobe with normal right thyroid lobe. The patient then underwent radioactive iodine ablation and was placed on thyroid hormone replacement. He has since been disease-free for 6 years.

*Case 2*

**Fig 3:**
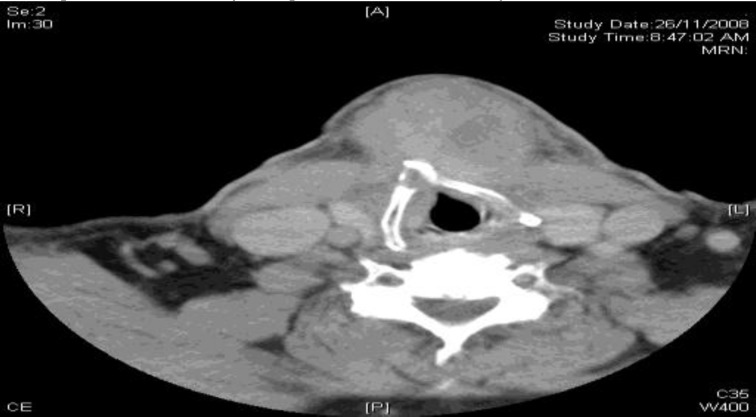
Axial CT scan of the neck at the level of hyoid bone demonstrating ill-defined low attenuation rounded lesions with thick wall in the anterior neck

A 56-year-old Malay gentleman, with type II diabetes mellitus and hypertension, presented with a 1-month history of anterior neck swelling. Clinical examination revealed a non-tender cystic swelling measuring 3 × 4 cm at the level of the hyoid bone, extending inferiorly down to the suprasternal notch. This swelling moved up with swallowing and with tongue protrusion. A CT scan (Fig. 3) confirmed a midline cystic mass measuring 4.1 × 4.7 × 5 cm within the subcutaneous tissues and left strap muscles, but separated from a normal-looking thyroid gland. The main impression from the image was a thyroglossal duct cyst.

FNAC of the neck lesion was found to be benign as it showed blood cells with numerous pigment laden macrophages. A Sistrunk procedure was performed, intraoperatively found to have an 8×8×6 cm cystic mass, which was encapsulated, multilobulated and attached to the hyoid bone. The lesion and body of the hyoid bone were excised. Histopathological examination of the lesion revealed PTC and the specimen from the hyoid bone was normal. The patient underwent a total thyroidectomy, which was followed by radioiodine ablation therapy. He has remained disease-free for 3 years following therapy.

*Case 3*

A 32-year-old Malay lady presented with a 6-month history of a right-sided neck swelling. There were no other complaints. Clinically, there was a mobile, cystic and non-tender, 2 × 2 cm swelling at the right posterior triangle. Nonetheless, there was no swelling on the left side of the neck. Similarly, there was no palpable thyroid swelling, and examinations of the nose, nasopharynx, oral cavity, oropharynx, and larynx were unremarkable.

**Fig 4:**
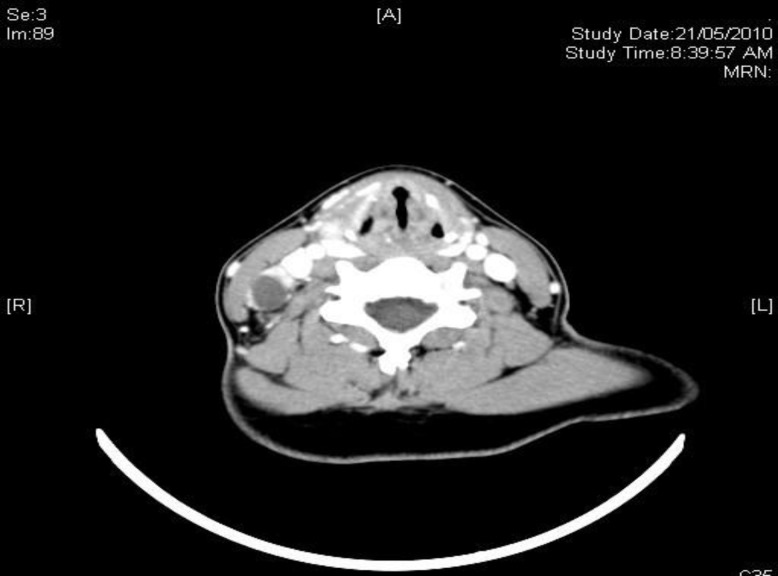
Axial CT scan of the neck demonstrating multiple cervical lymph nodes, with normal-looking thyroid gland.

A CT scan (Fig. 4) of the neck revealed multiple cervical lymphadenopathy bilaterally, with the largest measuring around 1.6 × 1.4 cm located at the right posterior cervical chain, which was enhancing the central necrosis. The thyroid gland was reported as normal.

A FNAC of the right posterior triangle swelling was performed and the finding showed true papillary configurations, monolayered sheets and singly dissociated atypical cells. The cells exhibited moderate-to-severe anisonucleosis, intranuclear grooves and intranuclear inclusions. 

The cytoplasm was dense, and conspicuous colloid was also seen in the background. Thus, an impression of metastatic papillary carcinoma was given.

**Fig 5:**
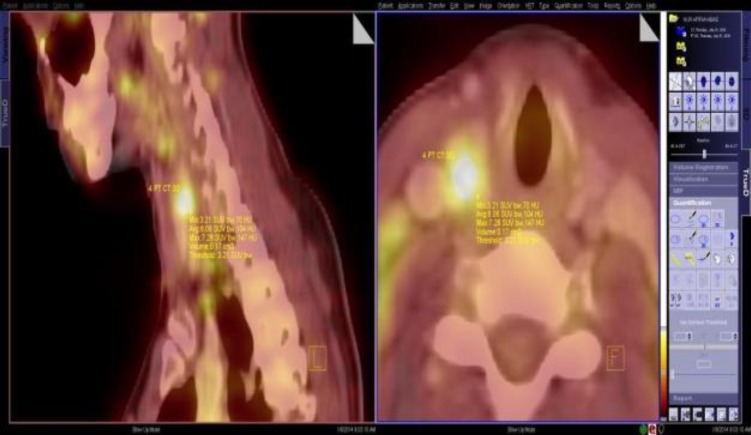
Reconstructed integrated PET/CT of the neck in sagittal (right) and axial (left) plane. Images demonstrated a focus of high 18F FDG uptake in the right lobe of the thyroid gland

The investigation proceeded with an ^18^F-FDG-PET CT scan (Fig. 5), which showed an increased FDG uptake in the superior part of the right thyroid lobe and the right-sided neck lymph nodes at levels III and IV. The patient underwent total thyroidectomy and radioiodine ablation therapy. She was then maintained on thyroid hormone replacement therapy and has since been disease-free for 2 years.

## Discussion

Cancer of the thyroid is the most common endocrine-related cancer, even though it is rare compared with other cancers, comprising less than 1% of all malignant tumors (5,6). Both PTC and follicular thyroid carcinoma (FTC) are differentiated thyroid cancers (DTC), which account for 90% of all thyroid cancers (7). Local lymphatic spread in PTC is common, occurring in 36–40% of patients, who usually present with a thyroid nodule and firm cervical lymphadenopathy. However, PTC can present clinically as a solitary cystic neck mass without any thyroid lesion. In our cases, there were no palpable lesions in the thyroid gland, which in turn lead to an incorrect initial diagnosis. Thus, it is important to note that this extrathyroidal neck cyst presentation may prolong proper diagnosis and treatment of the underlying malignancy as it could mimic benign cystic lesions of the neck such as a branchial cyst, thyroglossal duct cyst, dermoid cyst, epidermoid cyst, or cystic hygroma (8). A high index of suspicion toward malignancy should always be present after considering patient risk factors, especially taking into consideration their age (>40 years old) and history of radiation exposure to the neck. Thus, initial evaluation of an adult patient who presents with a cystic neck mass should include a detailed clinical history, a thorough head and neck examination, as well as FNAC analysis and imaging (9).

FNAC is considered the gold-standard investigation for the diagnosis of thyroid nodules. Various studies have shown that FNAC is a valuable diagnostic tool in the evaluation of solid neck masses with high sensitivity. However, its sensitivity is much less certain in cystic neck lesions. Clinicians must interpret FNAC results with extra caution and consider the use of an ultrasound-guided procedure (8–10). In our series, the FNAC was found to be negative in two out of three cases. If FNAC is negative for malignancy, an excisional biopsy should be considered. Imaging modalities such as ultrasound and CT are used to provide morphological information regarding the mass, as well as its extent and adjacent structures, including the cervical lymph nodes. While an ultrasound scan is the best imaging modality for delineating thyroid tumors, a CT scan has greater sensitivity for the detection of lymph nodes and extrathyroidal tumor extension (8).

Retrospective studies have reported incidences of incidental thyroid lesions, or thyroid ‘incidentalomas’, which were picked up by FDG-PET as part of the cancer metastases evaluation or screening in healthy individuals at a rate of 2–4%, with a malignancy risk of 27–37% in focal lesions (11,12). Such cases should be promptly evaluated with thyroid stimulating hormone (TSH) detection tests and sonography-guided FNAC as per guidelines (13), and subsequent surgery if indicated.

The incidence of cases of thyroid cancers is increasing yearly. This may be due to early detection by clinical examination and the use of imaging techniques such as neck ultrasonography and, more recently, the use of ^18^F-FDG-PET. ^18^F-FDG-PET CT is well recognized in cancer imaging. In addition to its high sensitivity in detecting most primary and secondary metastatic lesions, this imaging modality is useful in guiding clinicians in their management strategy and encouraging better decisions. In particular, patients in Case 1 and Case 3 benefited diagnostically from a ^18^F-FDG-PET CT scan, where uptake was shown not only in the clinically palpable neck nodes, but also in the affected areas of the thyroid gland, which were reported as normal in the CT scan.

Surgical management of papillary thyroid cancer involves a multifaceted treatment approach. However, controversies centering around both the extent of thyroidectomy and neck dissection (14–16) still persist until today. Currently, there are no clear guidelines for the management of cystic neck mass (8). Data from systematic review and meta-analysis showed that for patients with thyroid cancer of size >1 cm and <4 cm, without extrathyroidal extension and lymph nodal involvement, the initial surgical procedure could be either a near total or total thyroidectomy or lobectomy (15). Thyroid lobectomy alone is a sufficient treatment for small unifocal intrathyroid carcinoma in the absence of prior head and neck radiation, familial thyroid carcinoma or clinically detectable cervical nodal metastases (15). In fact, thyroid lobectomy alone may be sufficient for low-risk papillary and follicular carcinomas (15). Two of the patients in this study had excision of the lateral neck cyst and histopathology confirmed as a PTC, whereas in Case 3, the FNAC of the cystic neck mass demonstrated features of PTC. Thereafter, all three patients underwent thyroidectomy, radioactive iodine ablation and thyroid hormone replacement therapy. According to several recent studies, prophylactic neck dissection has shown no improvement in long-term patient outcomes (15,16).

## Conclusions

In conclusion, PTC can possibly present as a cystic neck mass, a presentation which is usually related to a benign lesion. This case series emphasizes that patients who present with a solitary cystic neck mass must be treated with a high index of clinical suspicion. Although it is not a first-line imaging modality, ^18^F-FDG-PET can be highly useful in assessing patients with a cystic neck lesion, where diagnosis remains uncertain even after standard investigations such as ultrasonography and FNAC have been performed.
